# The Resilience of Social Service Providers and Families of Children With Autism or Development Delays During the COVID-19 Pandemic—A Community Case Study in Hong Kong

**DOI:** 10.3389/fpsyt.2020.561657

**Published:** 2021-01-22

**Authors:** Paul Waiching Wong, Yanyin Lam, Janet Siuping Lau, Hungkit Fok

**Affiliations:** ^1^The Department of Social Work and Social Administration, The University of Hong Kong, Hong Kong, China; ^2^The Faculty of Social Sciences, The University of Hong Kong, Hong Kong, China

**Keywords:** children with autism or development delays, Hong Kong, COVID-19 pandemic, social service providers, service needs

## Abstract

**Background:** Hong Kong is one of the earliest cities to have hampered by the COVID-19. When preventive public health measures are enforced, specific groups, who have already been facing inequality before the outbreak, are likely to become more overlooked and vulnerable.

**Aim:** This community case study aims to describe the additional needs of families of children with autism spectrum disorder and other developmental issues, as well as unexpected difficulties and challenges social service professionals encountered when delivering service and their solutions toward these challenges.

**Methods:** A focus group with 10 professionals providing the Caregiver Skills Training Program was conducted.

**Results:** Poor families of vulnerable children were found to be challenged, more than average, in finding daily necessities during the initial stage of the outbreak. Most vulnerable children displayed additional problematic behaviors and emotional problems during the quarantine. The social service professionals addressed the family needs by providing tangible resources and offering online training, workshops, and programs to meet their needs. Several important lessons were learned. First, technology know-how on conducting online training, workshop, and program could be a challenge to some social service professionals and the parents. Second, the professionals reported that they made huge efforts to produce guidelines in protecting services users' privacy, to equip themselves with necessary skills in executing privacy-protection measures, and to keep exploring for safer alternatives. Third, providing tele-services in online mode represented a different interaction pattern between social service professionals and service users, especially in the recruitment processes and group dynamics.

**Conclusion:** In comparison with other cities, Hong Kong has responded to the COVID-19 efficiently and effectively based on the citizen's strict adherence to behavioral advice and the innovative altruistic efforts from the multi-sectors in the community.

## Introduction

As of May 13, the coronavirus disease 2019 (COVID-19) has infected more than 4 million people and claimed almost 300,000 lives worldwide (1–3). Hong Kong's first COVID-19 case was announced on January 23, 2020 (4). The experience of the avian influenza in 1997, severe acute respiratory syndrome (SARS) in 2003, particularly, and influenza A (H1N1) pandemic in 2009 has reinforced policy makers and the public to quickly adapt to many preventive public health measures to combat the COVID-19 pandemic. As one of the earliest cities to have hampered by the COVID-19, Hong Kong has been very successful in reducing community transmission by 44%, measured by the average number of people each infected person infects, or R (5), and among the 7.5 million people, the number of confirmed cases remained at 1,047 with four deaths as of May 13, 2020. As the world emerges from the COVID-19 pandemic, a key lesson to be learned is the “slow burn of injustice,” with avoidable health inequalities exposed by epidemics (6). Specific groups who have already been facing inequality before the outbreak are likely to become more overlooked and vulnerable. The aim of this community case study is to describe the contextual factors that foster the development of the resilience of the social service providers in helping vulnerable families and their children with special learning needs during the pandemic. As stated by many epidemiologists, there will be more pandemics to come, and this case study may have important prevention implications in the future pandemics.

## Background and Rationale

### The Pathways of Hong Kong in Becoming an “Experienced” City in Dealing With the Virus Outbreak

Hong Kong is an international and affluent city with an area of 1,106.8 km^2^ sustaining a total population of more than 7.5 million. The population density of Hong Kong stood at 6,930 persons per km^2^, and the most populous district achieved a density of 61,560 persons per km^2^ in 2019 (7, 8). Albeit having $382,046 GDP per capita, the Gini coefficient of 0.539 indicates that there is a significant wealth gap within the community (9, 10). Hence, >20% of the population are living under the poverty line (7). With such disparity, many of the poor families who are single parent and with lower education level have to rely solely on governmental resources and nongovernment organizations (NGOs) for various health and social services (7–14).

### Health and Social Services in Hong Kong Before the Pandemic

In Hong Kong, it was estimated that the incidence of autism spectrum disorder (ASD) is at 5.49 per 10,000, and the prevalence rate of ASDs is at 16.1 per 10,000 for children <15 years old (15). According to the government's recent mental health review, ASD was the main type of mental disorders among young children, comprising >60% of caseload of the child and adolescent services in public hospitals in 2015–2016, and the number of children with ASD seeking medical services from public hospitals had doubled between 2011 and 2016 (12).

Generally, the government has provided various support from early diagnosis to medical intervention and education. Through allying various institutions such as child assessment center, social welfare department, and education bureau (EDB), the government aims to improve the well-being of children and adolescents through developing a holistic support system. According to the EDB, the services for children with ASD cover assessment and identification, training and intervention, family support services, home-school cooperation, cross-sector collaboration, public education, and counseling and consultation (13). Many of these services are in “face-to-face” format, and the waiting time to receive any assessment through certified governmental agencies is, on average, 13–19.6 months (13).

### Health and Social Services in Hong Kong During the Pandemic

In January 25, 2020, the Hong Kong Government had raised the response level under the “Preparedness and Response Plan for Novel Infectious Disease of Public Health Significance (the Plan, hereafter)” to the emergency level. This plan was developed after the SARS epidemic in 2003 to allow Hong Kong to be much more prepared for future epidemics (16). The main goal of the plan is to ensure that a well-planned and fully integrated emergency management response can be implemented by all bureaus of the Hong Kong government with the support of the multisectors in the society.

The plan includes three response levels: alert, serious, and emergency. These response levels are based on risk assessment of the novel infectious disease that may affect Hong Kong and its health impact on the community. Emergency response level corresponds to a situation where the risk of health impact caused by the novel infection on local population in Hong Kong is high and imminent. Generally, it depicts a high risk of serious human infections caused by the novel infectious agent in Hong Kong, and serious infections may be widespread. It generally applies to situation when there is evidence or imminent risk of sustained community level outbreaks.

Accordingly, since late January, several preventive public health measures including surveillance, quarantine, social distancing, the use of face masks, and school closures have been implemented to suppress the transmission of COVID-19. On January 25, the education bureau announced the deferral of class resumption after Chinese New Year holiday for all schools, which marked the beginning of school suspension in response to the COVID-19 development in Hong Kong until further notice (16). Many nonurgent health care and social services were delayed or reduced.

### The Psychological Impact of Pandemic

Previous studies found that the outbreak of a novel virus was associated with the onset of psychiatric symptoms in mentally healthy individuals, exacerbated conditions of individuals with mental illness, and elevated burden for caregivers (17). The anxiety, fear, and stress experienced by the general public was associated with strong sense of insecurity, triggering off widespread panic buying of food and other basic necessities in at least two international cities (2). The prolonged closure of public services, quarantine, and impaired economic and social activities at the later stage further worsen the situation. A worry for further spread of COVID-19, distrust toward the government in their ability to contain the outbreak, anticipated economic downturn, and increased unemployment rate are associated with intensified negative emotions in the society (18–20).

#### Families of Children With Special Education Needs Under the COVID-19 Outbreak

Under such a problematic situation, parents had to handle multiple stressors simultaneously. Many parents struggle to secure enough resources, such as food and masks, to ensure home schooling of their children, taking care of the elderly, and going to work without contamination of their household (21). Limited data in the United States suggested the COVID-19 outbreak negatively affected vulnerable families more, including lower-income families and families of children with ASD (22).

Under the COVID-19 outbreak, families of children with ASD might face a particular difficult situation for at least three reasons. First, because of the ASD condition of their children, the parents might not be able to obtain enough tangible necessities. In particular, the closure of schools and child day-care centers shifted back the day-to-day caretaking role back to the parents while they were running here and there to fetch all kinds of daily necessities (2). Having limited patience and various vulnerabilities, children with ASD could not line up in the queue for long. As a result, these families often failed to get the necessities. The constant lack of resources causes stress and tension within families of children with ASD. Second, numerous research already showed that parents of children with ASD often suffered from elevated stress (23, 24), lowered quality of life (25), and heightened psychological distress (26). Third, because of the rigidity nature of people with ASD, the heavily disrupted daily routine has negatively affected their well-being (21).

Realizing the needs of parents of children with ASD, social service professionals fight against the odds to offer continued support and services for these families. Theoretically, social service professionals underwent the processes of resiliency as service providers (27–29). Resiliency of social service professionals can be conceptualized as the dynamic process in which social service professionals work with service users in encompassing positive adaptation within the context of significant adversity (29). In the time of COVID-19, Hong Kong social service professionals adopted a strength-based approach to mobilize community resources and empower service users to address their needs (27). It is necessary to document and summarize Hong Kong social service professionals' innovation, practice wisdom, and lessons learned for at least four reasons. First, “COVID-19 is not the first virus to threaten humanity, and it will not be the last” (30). The Hong Kong social service professionals' experiences can help to develop the practice guide and conceptual model for the future. Second, studies on social service professionals' view on the families of children with ASD under the period of COVID-19 pandemic are scarce (21). The document fosters the understanding of experiences of the families of children with ASD and serves as an expression of concern of academia toward these families in the time of uncertainty. Third, some service users mistakenly perceived that social service professionals might not be able to provide any kind of services in the period of COVID-19 outbreak. Our documentation helps to make social service professionals' work and the related challenges more visible and accountable (31). Fourth, up to date, parenting-related studies under the period of COVID-19 pandemic only present scholars' views [e.g., (21, 30)]. Little is known from frontline practitioners' perspectives. The current study can address this gap.

Based on a focus group interview with the social service professionals serving families of children with ASD, the current study aims to address the following research questions:

1. What are the needs of families of children with ASD and other developmental issues under the period of COVID-19 pandemic?
2. What are the services provided to families of children with ASD and other developmental issues under the period of COVID-19 pandemic?
3. What are the challenges social service professionals encountered?
4. What are the solutions to these challenges?

## Methodological Aspects

### Study Design

The current study adopts a descriptive qualitative research approach. Ten social work and psychological professionals were invited to join a semistructured interview. From their sharing, the needs of families of children with ASD as well as social service professionals' innovative response, practice wisdom, and lessons learned in the period of COVID-19 outbreak were summarized.

### Participants

The participants were mostly female (90%) and comprised clinical psychologists (30%), educational psychologists (10%), senior social workers (30%), registered nurses (20%), and early childhood educators (10%) from five local NGOs and two hospitals and the University of Hong Kong. Regarding education level, one (10%) completed a bachelor's degree, six (60%) completed a master's degree, and three (30%) completed a doctorate degree. All of whom have 7–15 years of experience serving families of children with ASD and developmental issues (Table 1).

**Table 1:** Background information of master trainers in Hong Kong.

**Profession**	**Gender**	**Years of experience**	**Education Level**
Clinical Psychologist	M	15	Doctor of Psychology (Clinical)
Clinical Psychologist	F	9	PsyD in Clinical Psychology
Clinical Psychologist	F	7	MSSc in Clinical Psychology !!break Master of Philosophy in Psychology
Counselling Psychologist	M	6	MSSc (Counselling) in Social Work
Counselling Psychologist	F	2.5	MSSc (Counselling) in Social Work
Educational Psychologist	F	Unknown	PhD. with specialization in Educational Psychology
Registered Social Worker	F	15	Master in Applied Psychology (Special Learning Needs)
Registered Social Worker	F	5	MSSc in Social Work
Registered Social Worker	F	12	Bachelor of social work
Nurse	F	11	Master of Nursing
Nurse	F	Unknown	Master of Nursing
Early Childhood Educator	F	7	Master in Early Childhood Education

All of the participants were the master trainers from the World Health Organization Caregiver Skills Training Program (WHO-CST, or CST) in Hong Kong. The program, which was adopted to the context of Hong Kong in 2018, aims to train caregivers of children 2–6 years of age with developmental disorders or delays, to provide better care for themselves and their children. To deliver CST locally, master trainers participated in training conducted by WHO. Four days were spent on learning the theoretical content of the program, and more hours have been spent on real-life practices in delivering program content, in order to reach the fidelity standards of the program. The program was originally designed for master trainers to deliver nine sessions and conduct three home visits in person, with each session comprising taught content, discussion, and role-play, lasting for 3 h on average. Each home visits involves observing play and home interaction between parent and child as well as master trainers demonstrating CST skills to enhance the interaction. Each visit lasts for about 1 h.

The master trainers from CST are chosen as the participants in this interview for several reasons. First, the implementation of CST in Hong Kong belongs to a large-scale community-based research program. The first phase of the research program reviewed the family needs and existing services for families of children with ASD in Hong Kong. Therefore, the master trainers are familiar with the situation of the families of children with ASD and the social services available for these families. Second, the master trainers are representatives from large leading NGOs and hospital authority from the government. The master trainers represent a wide range of social service professionals serving families of children with ASD in Hong Kong. Third, each master trainer supervises several facilitators, including parents of children with ASD, nurses, social workers, teachers, medical doctors, and occupational therapists. They are well-informed of the different aspects of life of families of children with ASD in Hong Kong. Fourth, the COVID-19 outbreak, especially the quarantine, discourages the open recruitment of participants for the program because the social service professionals are busy with restructuring their services. The master trainers from CST are the available experts ready for addressing research questions stated.

### Data Collection Process

The focus group interview was conducted through a teleconferencing application, during which participants were prompted to discuss the general effects that the pandemic poses on the parents and children with ASD and other developmental issues, services delivered and challenges they currently face, and their plans for providing services if the pandemic lasts for more than 3 months (see Appendix I in Supplementary Material). Responses were video recorded, transcribed by a research assistant, and sent to participants for checking accuracy.

### Analysis

For the current study, the second author read through the transcript of the focus group several times and summarized the initial themes generated from the transcript. The initial themes, then, was cross-checked by the first author to ensure the objectivity of these themes (see Appendix II in Supplementary Material for the list of themes). A trained research assistant coded the transcripts by using the coding scheme developed by the second author. The interrater reliability for the focus group was 0.91. The research assistant then counted the raw codes of each theme to further ensure that the data presented social service professionals' innovative response, practice wisdom, and lessons learned (32).

## Results and Discussion

### Needs of Families of Children With ASD—Tangible Resources

To facilitate the understanding of social service professionals' innovative response, practice wisdom, and lessons learned during the period of the COVID-19 outbreak, it is essential to introduce the needs of families of children with ASD as the basic context of the services provided. Based on the data from the semistructured interview, there were two major needs identified—(a) tangible resources and (b) intangible services. Nearly all the participants mentioned that many families of children with ASD needed tangible resources. Parents were desperate for surgical masks and alcohol-based hand rub in the initial stage of the outbreak. In February 2020, a panic buying of surgical masks has gone unresolved for more than 30 days (33). The panic buying of surgical masks could affect families of children with ASD more than the general public because many of these parents could not queue up for buying masks because of their children's conditions (22). As one of the professionals recalled: “…in the first week, they really would in the first week. Lining up everywhere like crazy.”

Also, the professionals also mentioned that some families of children with ASD required electronic devices in order to participate in online learning activities during school closure because of the spirit of “suspending classes without suspending learning” (34). However, many poor families of children with ASD did not have any electronic devices to support online learning. In response, the professional advocated for donation of electronic devices from the general public and passed the donated electronic devices to these families so that children with ASD in lower-income families could attend online classes and completed their assignments.

### Needs of Families of Children With ASD—Intangible Services

In addition, professionals also reported that children with ASD in Hong Kong displayed more problematic behaviors and emotional problems during the quarantine. This was consistent with previous literature on health emergencies. Rothe et al. (35) found that violence in children increased when schools were closed. An increase in problematic behaviors and emotional problems could be attributed partially to four reasons. First, according to stress-diatheses models (36), the outbreak was an additional stressor to children with ASD and other developmental issues, eliciting more problematic behaviors and emotional problems. Second, quarantine reduced social interaction. Without social stimulation, children with ASD might regress on the social skills and self-control skills they previously learned (37). With lower level of social skills and self-control skills, children with ASD and other developmental issues might display more problematic behaviors in interpersonal contexts. Third, the energy spent was reduced during the social distancing period, causing lower sleeping quality. Lower sleeping quality, in turn, magnified problematic behaviors and emotional problems (38). Fourth, children felt extremely boring, and parents exhausted with means to stimulate and occupy children.

On the other hand, parents told the professionals that they concerned a lot about their children's academic performance because of school closure. In most Chinese societies, parents always emphasize on exceling in schooling and examinations as their children's top responsibility (39–41). Parents of children with ASD spent a significant amount of time on keeping their children's learning in progress. Adolescents with ASD who needed to attend a public examination faced a lot of stress because the schedule of the public examination and resumption of school were uncertain.

Corresponding to the above needs, the social service professionals provided intangible services to address these needs. For instance, the professionals offered an emotional coaching program based on (42) model to parents of children with ASD (42). The program aims to train parents' skills in managing their children's emotional problems. In particular, to ensure smooth implementation and delivery of the program, social service professionals would ensure that service users have functional electronic devices available and stable internet connection, and the smooth installation and a test run of the teleconferencing software prior to the program. In addition, shortening the session time was suggested as parents were often torn between roles at home. For example, one coaching session shrunk from 2-h duration to 1 h. A self-compassion practice has been conducted. Information about being aware of child's emotion has been taught. Besides the main teaching content, more online viable interactive activities, such as polling and group discussions, were incorporated to keep participants engaged. Online parenting workshop was also conducted to share with parents how to schedule children's learning and occupy their time. Similarly, the social service professionals provided online training, phone counseling, and reaching out service for children with ASD in different developmental stages.

On the other hand, the professionals also noticed that children with ASD had unexpected positive experiences during the quarantine. As children with ASD did not need to go to school, they were free from problems of school bullying (43). They experienced more positive affect and could concentrate on their study. Some of the professionals had to provide individual counseling to help them make sense of such unexpected experiences.

### Suggestions for Providing Services for Families of Children With ASD in the Period of the COVID-19 Outbreak

Through trial and error, the professionals summarized a procedure to provide services for families of children with ASD. They suggested that social service professionals should concentrate on providing tangible resources at the early stage of outbreak. It is because providing tangible resources served several important functions in the period of the COVID-19 outbreak. First, based on the literature of community work, providing tangible resources are the important mechanism to approach the potential services users and promote available and future services (44, 45). Second, the COVID-19 outbreak created social distancing, which in turn increased loneliness (18–20). Providing tangible resources is a way to show concerns and build rapport with families of children with ASD. This could raise the willingness of families of children with ASD to receive services and increase their compliance in the future. Besides, providing electronic devices was the essential step for serving families of children with ASD with lower income in a “non–face-to-face mode.”

After addressing the needs of tangible resources, the professionals tried to relieve the issues brought forth by the children's special needs using “non–face-to-face mode.” As mentioned, the professionals offered online emotional coaching program, online parenting workshop, online special needs training, and phone counseling. With experiences, the professionals started to realize that the timing of offering services is important, especially for children with ASD in preschool ages and their parents. The professionals recommended offering online physical exercise training for the children with ASD in preschool ages during the morning and offering online parenting workshop or parenting program for their parents in the afternoon.

Because of class suspension and social distancing, children with ASD in preschool ages did not need to spend a lot of energy in the daytime. Some of them skipped the afternoon nap, and thus, they might demand more attention from their parents than before. Their parents then became unavailable for online parenting workshop or parenting program. Offering online physical exercise training for the children with ASD in preschool ages could use up part of their energy, increasing the likelihood of afternoon nap. Also, previous literature suggested physical exercises could lower the stereotypical behavioral patterns of children with ASD (46, 47), reduce self-stimulation behaviors (48), and increase social behavior (49) and academic engagement (50).

The professionals also suggested consolidating tips and recommendations for parents of children with ASD and other developmental issues onto a single source (e.g., a government web). Otherwise, these parents could be overloaded by excessive information. Consistent with literature on information overload, parents could not process too much information and automatically filter information when they are overloaded, causing biased decision-making (51, 52).

### Difficulties and Lessons Learned

Our professionals encountered several difficulties and lessons learned in serving families of children with ASD in the COVID-19 outbreak. The difficulties and lessons learned included technology know-how, privacy issues, and adjustment in non–face-to-face mode of services.

#### Technology Know-How

Although tele–social service might not be a new practice to many practitioners (53), the “technology know-how” on conducting online training, workshop, and program continued to be a challenge (54). During the COVID-19 outbreak, many services turned into online mode. The professionals reported that they had to consult the Information Communication Technology (ICT) experts in their NGOs or self-learn to master the knowledge and skills in setting up online services. Similarly, they had to design and produce guidelines in written and video format to teach the services users how to use electronic devices. This phenomenon echoed the application of information technology in social service services as a challenge to practitioners (55).

The relatively low level of competency in using ICT in social services might root in the understanding that humanity has been deemed as a essential to the sector, and empathy is a core quality of the helping professionals; therefore, education emphasizes on humanity training while offsetting ICT skills education. Limited studies indicated that only small portion of programs in undergraduate and postgraduate levels incorporated training in the use of electronic communications for social service professional trainees [e.g., Reamer (56)]. Similarly, current research focused on application of ICT in distance learning of the social work or mental health professional program. Little was done on developing guideline and conceptual model of how to deliver psychosocial services using information technology.

To be better prepared in responding to future challenges, in-house training courses and mental health professional education at university should include information technology course as compulsory subject without offsetting the humane side and empathy of the helping professionals. Researchers should also spend effort in investigating theoretical model of online mental health services by referring to literature on online interpersonal interaction [e.g., Jones et al. (57)].

#### Privacy Consideration

In relation to providing services in online mode, social service professionals had the ethical responsibility to protect services users' privacy (56). The professionals reported that they spent significant efforts to produce guidelines in protecting services users' privacy, to equip themselves with necessary skills in executing privacy-protection measures, and to keep exploring various safer software and resources. All these works became more salient when new reports stating serious privacy violation increased; for example, the BBC reported on an incident where a university lecturer's Zoom session had been interrupted by footages of child abuse (58).

Past studies indicated that individuals might be more ready to self-disclose their personal details online than face-to-face interaction (59, 60). However, online psychosocial services could be risky for electronic breaches or hacking. Also, unscrupulous or insensitive group mates might record the interaction in the online program and share with others.

In term of practices, services heads or supervisors in NGOs should develop a detailed guideline in protecting services users' privacy before launching online services. Also, social service professionals should educate their services users the potential risks and importance of privacy when receiving online services. Besides, social service professionals should proactively protect services users' privacy and confidentiality in online services contexts (56).

#### Adjustment in Non–Face-to-Face Mode of Services

Providing services in online mode represented a different interaction pattern between social service professionals and service users. The first difference was in the recruitment process. There was self-selection in the recruitment process. Families with lower socioeconomic status who did not have an electronic device or did not feel comfortable in using technology would not join their services. The self-selection process might violate the concepts of fair access and equal opportunity of receiving services (56).

To ensure the fair access and equal opportunity, social service professionals should proactively reach out to potential service users, express empathy and concern to isolated families, equip potential service users with necessary devices and skills for online services. They could also plan and recruit participants for face-to-face services in advance before the quarantine ended.

The second difference was in group dynamics (61). Group mate interactions and professional-service user interaction could be different between face-to-face and online format (57). For instance, some service users lost their focus in paying attention with online services than face-to-face one. Practitioners needed to assign participants who were familiar with each other to a group rather than all unfamiliar participants to facilitate mutual exchange in the online parenting program (62). All these implied conducting online services requires additional skill sets. Peer coaching and continued professional development should be encouraged within NGOs to sharpen social service professionals' micro skills in conducting online services.

#### Evidence-Based Practice

Another issue was about evidence-based practice. The COVID-19 outbreak forced social service professionals to deliver services in online settings. For instance, the professionals organized online parenting workshop and program as well as individual counseling. However, effectiveness of these services in online format in Hong Kong is underresearched.

Social service professionals are professionally and ethically obligated to provide evidence-based services. Practitioners should cooperate with researchers to conduct more action research to provide initial evidences for delivering services in online format (63). The COVID-19 outbreak then could be perceived as an opportunity in to advancing evidence-informed online services for families of children with ASD and other developmental issues.

### Limitations of the Study

The current study faced several major limitations. First, the current study adopted a nonprobability sampling method. The participants were social services professionals in Hong Kong who were limited to the master trainers from CST in Hong Kong. Our results could be biased toward families of children with ASD, who have voluntarily come in contact with the professionals. Our results might not be generalized to other service users (e.g., elderly) and to other societies. Second, some findings were bounded to be culturally relevant and might not be applicable to non-Chinese contexts; for example, some parents may be overly concerned about children's academic performance and afternoon nap. Third, the COVID-19 outbreak has not ended yet in Hong Kong. The current study could not document further service needs, innovation, and lessons learned for helping families of children with ASD readjustment to nonquarantine life.

## Conclusion

In 2003, during the SARS outbreak, the WHO commented that Hong Kong was one of the hardest cities in the world to control an epidemic because of the territory's immense population density and fluid boundaries with neighboring areas. It was because it was the first time that an infectious disease hit Hong Kong in such pace and scale, many of us underestimated its risk, and the government was trying too hard to contain public's panic at that time, which led to delayed decisions on enforcing territory-wide preventive public health measures. Eventually, 299 people were killed by the virus due to the absence of contingency planning, poor interagency coordination, unclear chain of command, and unsatisfactory resource and supplies support contributed to confusion and hindered effective implementation of infection control (64).

The government had since rolled out regulations, enhanced preparedness and response plans, with strengthened precautionary mechanisms. The mobilization of the public health and hospital systems, coordination of interdepartmental responses, information dissemination, quarantine requirements, school closures, and efforts to reduce close contact in public spaces have all benefited from the SARS and swine flu experiences. In this COVID-19 pandemic, according to one of the commentaries published in *Nature*, it says “Hong Kong seems to have given the world a lesson in how to effectively curb COVID-19” (65). We believe that the success of the current situation in Hong Kong is not a coincidence. The past experiences of the virus outbreak in Hong Kong has made the policy makers; civil servants of all government departments; charitable organizations; professionals in health, education, social welfare, and business sectors; multiple sectors; and all citizens here much more resilience to such a worldwide natural disaster.

Inevitably, some vulnerable groups would still be overlooked and experienced additional difficulties more than the public. In view of the crisis situation to fulfilling the unmet needs of the vulnerable families, many NGOs and large companies have been providing vulnerable families with tangible supports, i.e., giving out masks, food, and financial aids; giving out second-hand computers and tablets with free Wi-Fi-access cards; and intangible supports, i.e., developing free resourceful psychosocial–educational materials and distributing through both the traditional media and social media platforms and conducting online peer-support groups for the caregivers. Some of the materials and groups are delivered in other Asian languages so that families with ethnic minority backgrounds could benefit as well.

The main lessons learned from this experience are to defend a highly transmittable disease in an overcrowded city efficiently and effectively. It seems that (1) individuals can adhere to behavioral advices with the sense of protecting the well-being of self and others; (2) communities with a wide range of business, education, health, religious, social welfare, and voluntary sectors can pull together tangible and intangible resources quickly, identify the most vulnerable correctly, and distribute the resources efficiently and sometimes innovatively; (3) when the city's top leadership can enforce policies forcefully but flexibly, a silver lining can exist; and (4) both community and the government should consolidate useful information onto on webpage, so not to overload the parents when they are already stressed out. Learning from our master trainers, the social service sector has tried their best to deliver their assistances, whether it is educational or therapeutic, through any means even if the mean, i.e., ICT, was unfamiliar to them.

Since June 2019, the mental health burden of the Hong Kong people during the social unrest had already been documented with the increased prevalence rates of suspected depression and posttraumatic stress disorder at 11.2 and 12.8%, respectively (66). The additional impacts of the pandemic on the psychosocial well-being on the community are yet to be examined. Both incidents have severely impacted the young people and their families in Hong Kong, especially those who were arrested during the social unrest, those who are graduating from schools or transiting to higher levels of education or to the workforce, and those who have special learning and health needs. In these challenging times, investments in youth mental health and supporting their caregivers may be the most cost-effective ones for the future of Hong Kong.

## Data Availability Statement

The raw data supporting the conclusions of this article will be made available by the authors, without undue reservation.

## Ethics Statement

The studies involving human participants were reviewed and approved by Human Research Ethics Committee of the University of Hong Kong. The patients/participants provided their written informed consent to participate in this study (EA1912063).

## Author Contributions

PW, YL, JL, and HF substantially contributed to the conception of the work, drafting different components of the manuscript and revising other components. All authors approved the submitted version of the manuscript and agreed to be accountable for all aspects of the work.

## Conflict of Interest

The authors declare that the research was conducted in the absence of any commercial or financial relationships that could be construed as a potential conflict of interest.
