# Supplementary material for: The Resilience of Social Service Providers and Families of Children With Autism or Development Delays During the COVID-19 Pandemic—A Community Case Study in Hong Kong
Source: Front Psychiatry. 2021 Jan 22;11:561657. doi: 10.3389/fpsyt.2020.561657 (PMC7862762; doi:10.3389/fpsyt.2020.561657)
Supplement: Supplementary file 1 [file Data_Sheet_1.docx]

Appendix I- Meeting interview guide

1. How does the current situation of COVID-19 affect children with SEN and their parents?
   1. Mental health of the children of SEN
   2. Learning progress of the children of SEN
   3. Problem behaviors of the children of SEN
   4. Workload of caregiving of the parents
   5. Mental health of the parents
   6. Intensity and frequency of conflict with the children
   7. Intensity and frequency of conflict with family members
2. There are news reports saying parental stressful is extremely high under COVID-19. Can you tell us why?
3. Under COVID-19, what are the needs of the parents of children with SEN?
4. What kind of family of children with SEN are mostly affected by COVID-19?
5. What kind of services, the parents of children with SEN need most under COVID-19?
6. What services do you provide?
7. How do the parents seek help?
8. What roles social workers or mental health professionals can play in the current situation of COVID-19?
9. What are the difficulties you face when you try to provide services?
   1. Is there conflict between services users and social workers or mental health professionals under COVID-19?
   2. Are material resources enough?
   3. Do you have any technical difficulties in providing non-face-to-face services?
10. What are your current solutions you used to handle these difficulties?
11. What is your plan for providing services to family of children with SEN if the situation of COVID-19 continues for at least 3 months more?

Appendix II- Summary of themes with examples

| Themes |  |  |  |
| --- | --- | --- | --- |
| Tangible resources | - “some families with less resources are still looking for tangible items” | - “… in the first week, they really would in the first week. Lining up everywhere like crazy.” |  |
| Children’s emotional issues | - “ When I asked the parents how they are doing these days, they said that they are with a Tyrannosaurus everyday…always throwing tantrums” | - “They said they saw their children’s emotion deteriorate” | - “(In a grocery store, a sales lady) tapped on the child’s shoulder to ask him to step aside…his reaction returned to his original response, that is to shout, kept shouting at the sales lady.” |
| Trouble with regular routines | - “there is nothing left to play, also the biggest effect is that they can barely take afternoon naps… because their energy has not been spent” | - “ Some parents shared that the children always wanted to play with their mom, but they don’t know what else could they play with anymore” |  |
| Difficulty with schooling and examinations | - “the helpless part is that when they don’t know (the answer), they could not ask help from their classmates or teachers” | - “Because we don’t know when will school resume, so for some ASD students, it’s a lot of anxiety…” |  |
| Unexpected benefit | - “ they can hide at home, they felt very comfortable” | - “when we contacted, we talked to him, he said ‘miss, I am very bored, I don’t want to play computer games, I want to go to school.’ He loved to play computer games but he doesn’t want to play anymore.” | - “think about it teenagers go to school where their classmates don’t like them, bully them, also involving lots of social issues but during class suspension, all of these stopped, so they think that this is really good.” |
| Difficulty in service delivery | - “we stopped (face to face) completely” | - “Zoom actually a lot parents are not willing to turn on their camera, only have sound like listening to a radio” | - “(Due to confidentiality issue with Zoom) I and my colleagues are still looking for other contingency” |
| Current solution for service delivery | - “We have a parenting group, which teaches emotional coaching skills. When we do it, we uses Zoom, the format will be a little different, the duration is different.” | - “we made videos to teach them how to download (Zoom) step by step. We also have text version to help them understand” | - “everything became Zoom…our ST went to do (their program through Zoom) and it’s okay, but the others haven’t responded” |
